# The Impact of a Technology-Assisted, Two-Month Breathing Exercise Intervention on Daily Affect, Weekly Stress and Examinational Stress

**DOI:** 10.3390/bs16050778

**Published:** 2026-05-14

**Authors:** Konrad Rudnicki, Glen Joris, Floortje Meyvis, Marthe Verschraegen, Karolien Poels

**Affiliations:** The Antwerp Social Lab, University of Antwerp, 2000 Antwerp, Belgium; glen.joris@uantwerpen.be (G.J.); floortje.meyvis@uantwerpen.be (F.M.); marthe.verschraegen@uantwerpen.be (M.V.); karolien.poels@uantwerpen.be (K.P.)

**Keywords:** breathing exercises, stress, ecological momentary assessment, academic stress, haptic biofeedback

## Abstract

Breathing exercises are a low-cost strategy for mitigating daily stress, yet their enhancement via haptic biofeedback devices remains under-explored. These devices may facilitate adaptive breathing by personalizing rhythms via providing haptic cues based on heart rate, blood oxygenation and heart rate variability. We conducted a 2-month longitudinal trial with 62 healthy participants ($M_{\mathrm{age}}=26.9\pm 8.1$ years; 81% female) to test whether a haptic breath pacer (“Moonbird”) augments the stress-reducing effects of breathing exercises. Participants were randomized to: (1) haptic feedback + app, (2) app-only, or (3) a no-exercise control. Ecological Momentary Assessment tracked daily affect (valence/arousal) and weekly stress. Students (n=45) also reported pre-examination stress. Between-subjects analyses revealed no condition effects on measured outcomes. However, within-subjects analyses showed that participants who felt higher arousal and valence but performed their breathing exercises felt a significant reduction in arousal and increase of valence on the following day. In the haptic group, these effects were moderated by user experience, indicating that benefits are contingent on device usability. Finally, highly stressed students who performed more exercises reported lower examination stress at the end of the semester, irrespective of their assigned condition.

## 1. Introduction

Stress has become a prevalent challenge to mental and physical health in modern life, fueling interest in low-cost, self-regulatory strategies for stress management. One such well-documented strategy is the practice of controlled breathing exercises (Morgan et al., 2025). Breathing techniques—ranging from slow diaphragmatic breathing to more structured “breathwork” programs—have been used in both clinical and non-clinical settings to induce relaxation and improve emotional well-being. A growing body of research supports the efficacy of breathing exercises in reducing stress and anxiety (Bentley et al., 2023; Garg et al., 2024; Li et al., 2023; Morgan et al., 2025). Recent meta-analyses and reviews conclude that deliberate breath regulation is associated with significant, albeit modest, reductions in self-reported stress levels and anxiety symptoms compared to no-treatment controls (Fincham et al., 2023; Leyro et al., 2021). For example, a meta-analysis of 12 randomized trials found that breathing-based interventions led to lower subjective stress (g≈−0.35) and improved mental health outcomes (Fincham et al., 2023). Similarly, a comprehensive review of 40 trials of “respiratory interventions” for anxiety reported moderate-to-large anxiety reductions, affirming that breathing-focused techniques can serve as effective standalone or adjunct treatments for anxiety disorders (Leyro et al., 2021). Such findings align with earlier systematic reviews indicating that diaphragmatic (deep) breathing produces beneficial changes in stress-related physiological markers (e.g., reduced cortisol, blood pressure) as well as psychological stress ratings (Hopper et al., 2019; Li et al., 2023). Overall, the literature converges on the notion that breathing exercises cultivate a parasympathetic “relaxation response” that counters the sympathetic arousal underpinning stress and anxiety (Bentley et al., 2023; Zaccaro et al., 2018). Breathing practices are also generally safe, self-administered, and readily accessible, making them attractive as daily stress management tools.

The psycho-physiological mechanisms underlying these effects are increasingly well-understood. Slow, controlled breathing directly modulates autonomic nervous system activity by increasing vagal efference to the heart, producing characteristic increases in high-frequency heart rate variability (HRV) that indexes enhanced parasympathetic tone (Zaccaro et al., 2018). This vagal engagement is theoretically significant beyond its cardiovascular effects: according to the neurovisceral integration model (Thayer et al., 2012), vagally mediated HRV reflects the functional integrity of a central autonomic network linking prefrontal cortical regions to subcortical structures involved in threat appraisal and emotional reactivity, including the amygdala and hypothalamus. Higher vagal tone is therefore associated with greater top-down inhibitory control over stress responses, enabling more flexible and adaptive regulation of affect (Thayer et al., 2012; Zaccaro et al., 2018). Slow breathing may thus reduce stress and improve mood not merely by inducing momentary relaxation, but by transiently augmenting the neural regulatory capacity that helps affect regulation more broadly (Zaccaro et al., 2018).

Breathing-based interventions have demonstrated benefits across diverse populations and contexts. In healthy adults, even brief daily breathing sessions can yield improvements in mood and physiological relaxation. For instance, Balban et al. (2023) showed that a short, structured breathing practice each day over one month significantly enhanced positive affect and reduced autonomic arousal, outperforming a mindfulness meditation control condition. In student populations, breathing techniques have been integrated into stress-reduction programs with promising outcomes. A controlled trial among university students found that a breathing-based wellness program led to greater reductions in stress and improvements in positive affect than a conventional mindfulness course or an emotional intelligence training (Seppälä et al., 2020). Likewise, high school studies suggest breathing training can mitigate academic stress: Mahalakshmi et al. (2024) reported that daily deep breathing exercises helped decrease perceived exam-related stress in high school students. These examples illustrate the broad applicability of breathing exercises for improving daily affect balance and stress resilience. However, many prior studies have examined relatively short-term interventions or acute effects in laboratory settings. There is comparatively little data on the longitudinal impact of maintaining a breathing practice over multiple months in one’s day-to-day life. It remains an open question to what extent regularly practicing breathing exercises can produce cumulative benefits observable in everyday affective states and stress levels outside the laboratory. In particular, studies employing ecological momentary assessment (EMA)—i.e., repetitive sampling of participants’ affect and stress in real-world environments—are scarce in this domain, with only a handful of investigations having used EMA designs to track the effects of breathing interventions in daily life (Loo Gee et al., 2016; Perreau et al., 2023). Without such data, the stability and practical significance of breathing-induced stress relief in daily life are not fully understood. This gap in the literature points to the need for field-based trials tracking how breathing exercises influence people’s emotional well-being and stress in real time—and raises the question of whether emerging technology-assisted tools, from smartphone apps to haptic biofeedback devices, can support and enhance such practice in ways that translate to real-world benefit.

### 1.1. Technology-Assisted Interventions and the Role of User Experience

In parallel with research on breathing techniques themselves, there has been rapid growth in technology-assisted interventions designed to facilitate and enhance breathing exercises (Sîrbu & David, 2024). Mobile health and wearable technologies offer new ways to guide breathing practices, provide feedback, and potentially increase user engagement. Dozens of smartphone apps now teach paced breathing or mindfulness breathing exercises, and initial evidence suggests these can modestly improve stress outcomes. Numerous recent systematic reviews of stress-management apps found that app-based interventions targeting relaxation (including breathing and meditation apps) yield small but significant reductions in both psychological and physiological indicators of stress (Brinsley et al., 2025; Linardon et al., 2019, 2024; Loo Gee et al., 2016; Sîrbu & David, 2024). Breathing apps can deliver on-demand guidance, structured programs, and reminders, which may help users adhere to regular practice. Beyond apps, researchers and designers have introduced a variety of sensor-enabled devices to support breathing regulation. A systematic literature review by Honinx et al. (2023) surveyed the landscape of commercially available “meditation and breathing devices” for stress reduction, identifying over 40 unique devices. These range from wearables (e.g., chest straps, wristbands, earbuds) that monitor respiratory metrics to handheld gadgets and even huggable objects that guide breathing rhythms through different stimuli. The incorporated feedback modalities are diverse—including visual guides (lights or animations synced to inhale–exhale cycles), auditory cues (tones, music, voice instructions), and haptic signals such as vibration or gentle pressure (Honinx et al., 2023). Notably, this review concluded that while technology-assisted breathing tools are plentiful, the evidence base for their efficacy is limited. Most device concepts have not been rigorously evaluated in peer-reviewed studies, and few have been tested beyond short-term use. Moreover, Honinx et al. (2023) emphasize a need for more user-centered design in this space: many devices were developed without sufficient input from end-users and without clear grounding in user preferences or behavioral science. This highlights an important consideration that simply introducing technology into a breathing intervention does not guarantee improved outcomes—the tool must be acceptable and engaging to the user to positively influence their practice.

A growing body of evidence suggests that the usability, engagement, and overall user experience (UX) of digital health tools can substantially influence their therapeutic impact (Opie et al., 2024). Poor ease-of-use or a lack of engaging design often leads to attrition, limiting users’ exposure to an intervention and blunting its benefits (Opie et al., 2024). Conversely, interventions that prioritize an intuitive, user-centered design tend to see better adherence and even larger effect sizes in outcomes (Gan et al., 2021; Lemon et al., 2020; Wu et al., 2021). For example, Smyth et al. (2018) showed that user experience in a gamified app designed to alleviate depressive symptoms was the best predictor of whether that app successfully helped its users. Similarly, Rudnicki et al. (2025) have shown that positive user experience can be a gating factor for efficacy: in a randomized trial of brief biofeedback-assisted meditation, improvements in objective interoceptive accuracy were observed only among participants who reported a good UX with the meditation tool. These insights highlight that simply introducing novel technology is not enough—how users perceive and interact with the tool is critical to realizing its benefits. Accordingly, the present study will assess participants’ user experience (using the UMUX survey (Finstad, 2010)) and examine whether UX moderates the effectiveness of the guided breathing exercise, recognizing that an acceptable and engaging tool is likely a precondition for optimal outcomes.

### 1.2. Haptic Biofeedback

Among technology aids for breathing exercises, haptic feedback devices have garnered particular interest as a means to deliver breathing guidance in an intuitive, non-intrusive manner. Haptic breathing devices typically provide a rhythmic vibration or pulsation that users can feel and follow, matching their breath to the pattern of the vibration. The appeal of haptic breath pacing is that it allows users to close their eyes or divert attention from a screen; the body’s sense of touch carries the timing information for inhales and exhales. Initial research suggests that users appreciate and readily adapt to haptic breathing cues. In the review by Honinx et al. (2023), participants in various studies expressed a strong preference for devices with haptic feedback, yet the authors noted that this user preference has often been overlooked by device developers. Some early prototypes and commercial devices exemplify how haptic feedback can be implemented. Handheld “breath pacers” like the Moonbird™ (Moonbird B.V., Antwerpen, Belgium) or Core™ devices gently expand/contract or vibrate in the hand to signal breathing rhythm. In a recent mixed-methods trial, Honinx et al. (2025) evaluated these two haptic breathing devices (Moonbird™ and Core™) among highly stressed individuals. Both devices were found to acutely slow breathing rate and induce physiological signs of relaxation. Other innovative applications of haptic breathing support have been tested in context: for example, a system of vibrating wearables was used to guide car commuters through slow breaths during stressful driving, yielding reduced physiological arousal without impairing driving performance (Balters et al., 2020). Similarly, researchers have explored social robots that synchronize their movements (rising and falling) with a breathing cycle for users to mimic, as a way to reduce anxiety via deep breathing (Matheus et al., 2022).

While these pilot studies and prototypes demonstrate the feasibility and user acceptance of haptic breathing aids, their effectiveness in producing sustained stress-reduction benefits remains to be fully demonstrated. Do haptic biofeedback devices actually augment the well-known benefits of breathing exercises? Do these benefits persist beyond singular sessions and impact daily life? The current literature offers only tentative answers. Some findings imply that adding biofeedback or sensory cues could enhance outcomes by increasing users’ focus or adherence. For instance, Leyro et al. (2021) noted in their meta-analysis that respiratory interventions incorporating biofeedback tended to show larger anxiety reductions, though direct comparisons are few. Conversely, a controlled experiment by Laborde et al. (2022) found that slow breathing yielded similar short-term emotional benefits whether or not real-time heart-rate biofeedback was provided, suggesting the breathing practice itself was the active ingredient. Clearly, more empirical study is needed to isolate the value of technology enhancements such as haptic feedback in the context of stress-reduction breathing training.

In summary, breathing exercises are an evidence-supported method for managing daily stress and enhancing affective well-being. Modern technological tools—from mobile apps to wearable breath pacers—offer new opportunities to support breathing practices, but their added benefits and optimal design features are still being investigated. One notable knowledge gap is the lack of longitudinal, real-world data on how practicing breathing with or without such devices influences everyday stress experiences. Most prior studies have assessed outcomes at a coarse pre-post level or in controlled environments, leaving unclear whether the momentary affective improvements from breathing exercises translate into lasting day-to-day stress reduction.

### 1.3. Examinational Stress

There is also little research focusing on academic or examinational stress in students using breathing interventions, despite some preliminary evidence that breathing techniques can help students cope with exam anxiety (Bentley et al., 2022; Mahalakshmi et al., 2024). The limited research on breathing interventions for academic or examinational stress represents a significant gap in the stress management literature, particularly given the unique ecological validity that examination periods offer for studying acute stress responses in naturalistic settings. While breathing exercises are theoretically well-positioned as interventions during moments of elevated stress, due to their immediate accessibility and physiological effects on the autonomic nervous system, few studies have systematically examined their application in the high-stakes, time-constrained context of academic examinations. The existing evidence base consists primarily of small-scale trials with mixed findings: Rosenberg and Hamiel (2021) found that a biofeedback respiratory device significantly reduced test anxiety during actual exam periods compared to self-directed breathing or psycho-education (n=34), while Ortega-Donaire et al. (2023) demonstrated that guided breathing administered immediately before final nursing assessments improved knowledge scores among students with moderate anxiety. Technology-assisted approaches show particular promise, with Ovadia-Blechman et al. (2022) reporting large effect sizes for device-guided breathing training in reducing student anxiety, though their pilot study (n≈21) lacked sufficient power for statistical significance. The existing investigations remain constrained by their small sample sizes, heterogeneous intervention timing (ranging from chronic training protocols to acute pre-test administration), and limited real-time physiological monitoring during the actual examination scenarios.

### 1.4. The Present Study

The introduction above identifies three converging gaps in the literature: the absence of longitudinal, real-world data on how daily breathing practice affects momentary affect and stress over months rather than days; the unresolved question of whether haptic biofeedback devices add meaningful benefit beyond app-guided breathing alone; and the near-complete lack of EMA-based studies examining breathing interventions during ecologically valid stress episodes such as academic examinations. The present study was designed to address all three simultaneously. In a randomized controlled trial, over a period ranging from two to three months, we compared three groups: (1) a control group with no breathing exercises, (2) a group performing daily app-guided (Breathe™ app, Havabee, Mumbai, India) breathing exercises, and (3) a group using a combination of a smartphone app and a haptic breathing device (Moonbird™, Moonbird B.V., Antwerpen, Belgium) for daily breathing exercises. Crucially, we employed ecological momentary assessment (EMA) to track participants’ daily affect (valence and arousal) and weekly stress levels throughout the intervention, and for student participants we also measured their examinational stress on the days of their exams using EMA. This design allowed us to capture both between-group differences in overall stress reduction and within-person dynamics—for example, whether engaging in a breathing exercise on one day predicted improvements in mood or stress by the next day.

Based on the theoretical foundations and empirical evidence reviewed above, we formulated several specific hypotheses for the present investigation.

Given that meta-analyses demonstrate significant, albeit modest, reductions in self-reported stress and improvements in mental health outcomes from breathing-based interventions, and that even brief daily breathing sessions can improve mood and physiological relaxation in healthy adults:

**Hypothesis** **1** (Main effect of breathing practice). *We hypothesized that the participants in both breathing exercise groups (app-assisted and app + haptic device-assisted) would show greater improvements in daily affect (higher valence, lower arousal) and reduced weekly stress levels compared to the control group over the intervention period.*

Drawing on initial research suggesting that haptic feedback devices provide intuitive, non-intrusive breathing guidance that users appreciate and readily adapt to, and considering evidence that respiratory interventions incorporating biofeedback tend to show larger anxiety reductions:

**Hypothesis** **2** (Effects of haptic feedback). *We hypothesized that the addition of the haptic breathing device (Moonbird™) would augment the stress-reducing and affect-enhancing effects of app-guided breathing exercises on daily affect (valence, arousal) and weekly stress.*

Despite limited research on breathing interventions specifically targeting academic stress, breathing techniques are uniquely suited to help with acute, momentary stress events. Given that examinational periods represent a series of strong, personally salient stress episodes:

**Hypothesis** **3** (Examinational stress). *We hypothesized that participants in the breathing exercise groups would report lower examinational stress on the days of their exams.*

Given that breathing exercises function as stress-regulation tools that primarily benefit individuals experiencing elevated stress or negative affect (cannot lower stress when it is already low):

**Hypothesis** **4** (Next-day predictive effects). *We hypothesized that the effectiveness of breathing exercises on a given day would be moderated by current affect. Specifically, we expected that engaging in breathing exercises would predict improved daily affect (higher valence, lower arousal) on the following day primarily among participants who were experiencing worse mood or higher stress at the time of practice, reflecting the targeted regulatory benefits of breathing interventions for those who need them most.*

Based on evidence that usability and overall user experience substantially influence the therapeutic impact of digital health tools, with positive user experience serving as a gating factor for intervention efficacy:

**Hypothesis** **5** (User experience moderation). *We hypothesized that user experience would moderate the effectiveness of the breathing interventions. Specifically, we expected that participants with more positive user experience ratings would show greater stress reduction and affective improvements.*

By testing these hypotheses through ecological momentary assessment in the participants’ natural environments, this study aimed to provide preliminary evidence for the real-world efficacy of technology-assisted breathing interventions and identify key factors that optimize their stress-reducing benefits in daily life contexts.

## 2. Materials and Methods

### 2.1. Participants

Sixty-two participants ($M_{\mathrm{age}}=26.9\pm 8.1$ years; 81% female) were recruited via the internal student platform and mailing list of the University of Antwerp. Forty-five participants were students and could provide examinational stress surveys. An inclusion criterion was introduced where potential participants were only recruited if they agreed with the statement: “I experience stress in my daily life.” This criterion was used to limit the potential ceiling effects if we happened to recruit people whose stress from daily life could not further benefit from breathing exercises. Potential participants were excluded if they self-reported regular spontaneous engagement in breathing exercises leading up to the study, as the control group was expected not to perform any. Potential participants were also excluded if they self-reported suffering from any anxiety disorders. The participants were compensated for their participation by entering a raffle after the study concluded, where they each had a 1/3 chance at winning one of the haptic feedback breathing devices. Every participant provided written informed consent prior to participation.

This study was sponsored in part by the Moonbird™ company, which provided 60 haptic breathing exercise devices to the University of Antwerp free of charge. Moonbird™ is a commercial Belgian startup that created these devices. Moonbird™ had no role in the study design, data collection, data analysis, data interpretation, or writing of the report.

The design and protocol of this study was approved by the Ethics Committee for Social Sciences and Humanities of the University of Antwerp (SHW_2024_224_1).

### 2.2. Design and Intervention

A randomized controlled trial was performed with three experimental conditions:

1. An app-based box-breathing exercise group, which was instructed to perform daily breathing exercises with a dedicated application (Breathe™).
2. An app + haptic feedback box-breathing exercise group, which was instructed to perform daily breathing exercises with a dedicated application and a device (Moonbird™).
3. A control group that was not subjected to any breathing exercises nor any other intervention.

The participants were randomly assigned to one of the three groups during an onboarding session where the study protocol was explained, devices were provided (if applicable) and a practice 6 min breathing session was performed to ensure that the participants had been properly instructed in how to use their apps and devices.

The study lasted between 2 and 3 months. Recruitment began on 28 March and concluded on 30 April. All participants ended the study on 30 June, which means that the intervention time ranged from 61 days to 95 days. Average intervention time was: $M_{\mathrm{time}}=86.9\pm 9.4$ days.

The app-based breathing exercise group was equipped with the Breathe™ app and instructed to perform at least 6 min of the built-in box-breathing exercise every day for the duration of the study. The app + haptic feedback breathing exercise group received the same instructions and performed identical box-breathing, but with the aid of the Moonbird™ devices. At 6:15 PM each day, both groups received a reminder to perform the breathing exercise.

Each session comprised a 6 min box-breathing sequence delivered via the app (and, when assigned, synchronized haptic cues). Participants were instructed to sit upright, place the handheld device in their dominant hand, and follow a 4 s inhale → 4 s inspiratory hold → 4 s exhale → 4 s expiratory hold rhythm (≈4 breaths per minute). Continuous guidance from the apps ensured ∼23 complete cycles, totaling 360 s of controlled ventilation.

In the app-based group, the Breathe™ app displayed an expanding/contracting circle, which paced each phase of the abovementioned box-breathing cycle. Additionally, participants were guided by either a male or female voice narrating the instructions: “breathe in,” “hold,” and “breathe out” at appropriate times. An ambient background sound played during the narration, with participants able to choose between four options in the app settings: sea (the default option), dawn, stream, or rain shower. The app was selected for three reasons: (1) it delivered the same box-breathing protocol (4 s inhale, 4 s hold, 4 s exhale, 4 s hold) as the Moonbird™ device, ensuring the breathing technique itself was held constant across both active conditions; (2) it provided verbal narration guiding the users through each phase of the cycle, which was considered necessary given that the participants were naive to breathing exercises at the time of recruitment; and (3) its visual pacing interface—an expanding and contracting circle—reflects the most common design approach in breathing apps, making it a representative choice as an app-only comparator.

In the app + haptic feedback group, the Moonbird™ app displayed the remaining session time and allowed users to disable the audio guidance, if preferred. In addition, the app presented real-time physiological data, with participants able to select and switch between three metrics during each session: heart rate (HR), heart rate variability (HRV), or coherence. HR was measured via photoplethysmography (PPG) using a sensor embedded in the Moonbird™ device, which detected fluctuations in blood volume through the thumb. HRV was calculated from the variation in time intervals between successive heartbeats, derived from the same PPG signal. Coherence was computed as the degree of synchronization between heart rhythm and respiratory patterns, reflecting the regularity and sinusoidal structure of the HRV waveform. At the end of each session, the app provided a visual summary of the recorded physiological metrics.

### 2.3. Measurements

Daily affect was operationalized with arousal and valence, measured with an adapted version of the Self-Assessment Manikin (Bradley & Lang, 1994). The original SAM employs a 9-point pictorial rating scale; for the present study, this was replaced with a visual analogue scale (VAS) ranging from 0 to 100. This adaptation was made for two reasons. First, the continuous format provides higher measurement resolution, which is particularly important in intensive longitudinal designs where the analytic goal is to detect fine-grained within-person fluctuations rather than broad between-person differences. Second, a slider-based VAS is better suited to smartphone delivery than the original pictorial manikin format. The underlying valence and arousal dimensions remain unchanged from the original instrument. Arousal ratings capture the perceived intensity of physiological activation—ranging from relaxed or sleepy to highly excited. Valence ratings index the degree of positive versus negative affect and predict approach vs. withdrawal motivational tendencies (Bradley & Lang, 1994).

Adherence to the assigned breathing exercises was measured using a single item: “Did you perform the breathing exercises yesterday?” Asking about the preceding day was done to ensure that the time of administering the surveys did not affect the adherence data. If the item had asked about the current day, it stands to reason that it could have reached the participants before they performed their planned exercise for the day.

Weekly stress was measured using the validated 30-item Daily Stress Response Scale (Debowska et al., 2022), which contains 15 items for psychological stress (e.g., “I have felt upset”) and 15 items for physiological stress (e.g., “My heart has been racing”), which were rated on a 5-point scale ranging from 0 (Never) to 4 (Almost All The Time).

Examinational stress was measured using selected items from the validated Examination Stress Scale by Sung and Chao (2015). The scale was originally constructed to measure trait examinational stress—a general tendency the person had towards feeling stressed before exams. We have selected the 10 items from the “Anxiety Responses,” which were rephrased to refer to the present moment (e.g., “I experience stomach discomfort before or during examinations” was rephrased to: “I experienced stomach discomfort”—where each item was qualified with: “Before or during today’s exam...”). The items were rated on a 5-point scale ranging from 0 (Totally Disagree) to 4 (Totally Agree).

User experience was measured with the Usability Metric for User Experience (UMUX) by Finstad (2010). UMUX consists of four items measured on a 7-point scale ranging from 0 (Totally Disagree) to 6 (Totally Agree):

1. The capabilities of Breathe/Moonbird meet my requirements.
2. Using Breathe/Moonbird is a frustrating experience.
3. Breathe/Moonbird is easy to use.
4. I have to spend too much time correcting things with Breathe/Moonbird.

Participants’ answers in this questionnaire were re-coded following the procedure by Finstad (2010). After re-coding, UMUX yields values between 0–100, where higher scores indicate better user experience.

Ecological Momentary Assessment. Participant’s daily arousal and affect, weekly stress and examinational stress were collected using an EMA service (m-path (Mestdagh et al., 2023)) installed on participants’ phones during the onboarding session. The software sent the following notifications to the participants’ phones:

1. Daily, at a random time between 9:00 and 18:00, three survey items measuring adherence, arousal and valence. This survey was available to be filled out until 23:59 on the same day.
2. Weekly, on Sundays at 9:00, a 30-item survey measuring the subjective experience of stress during the past week (weekly stress). This survey was available to be filled out until 18:00 on the following Monday.
3. Once a month, at 9:00, a 4-item user experience survey measuring the subjective experience of using the specific app and/or device that participants were given (user experience). This survey was available to be filled out until 18:00 on the following day.
4. Once during the study, on 10th May, student participants received a survey asking them to fill in the planned dates of their exams.
5. On the days of the exams, at 8:00, student participants received a survey measuring the subjective experience of examinational stress (examinational stress). This survey was available to be filled out until 18:00 on the same day.

## 3. Results

Descriptive statistics of the study variables averaged across the whole intervention period are presented in Table 1. Details of the construction of regression models for statistical analysis are provided in Appendix A.

### 3.1. Between-Subjects Results

To test **H1** (the main effect of breathing practice on daily affect and weekly stress) and **H2** (augmentation by haptic feedback) at the group level, a series of linear mixed-effects analyses (equivalent to a mixed-factor repeated-measures ANOVA with random intercepts for participants; see Appendix A) examined whether *Daily Arousal*, *Daily Valence* and *Weekly Stress* differed between the three experimental groups (Moonbird™, Breathe™, Control), changed over time, or showed a Group × Time interaction.

**Figure 1:**
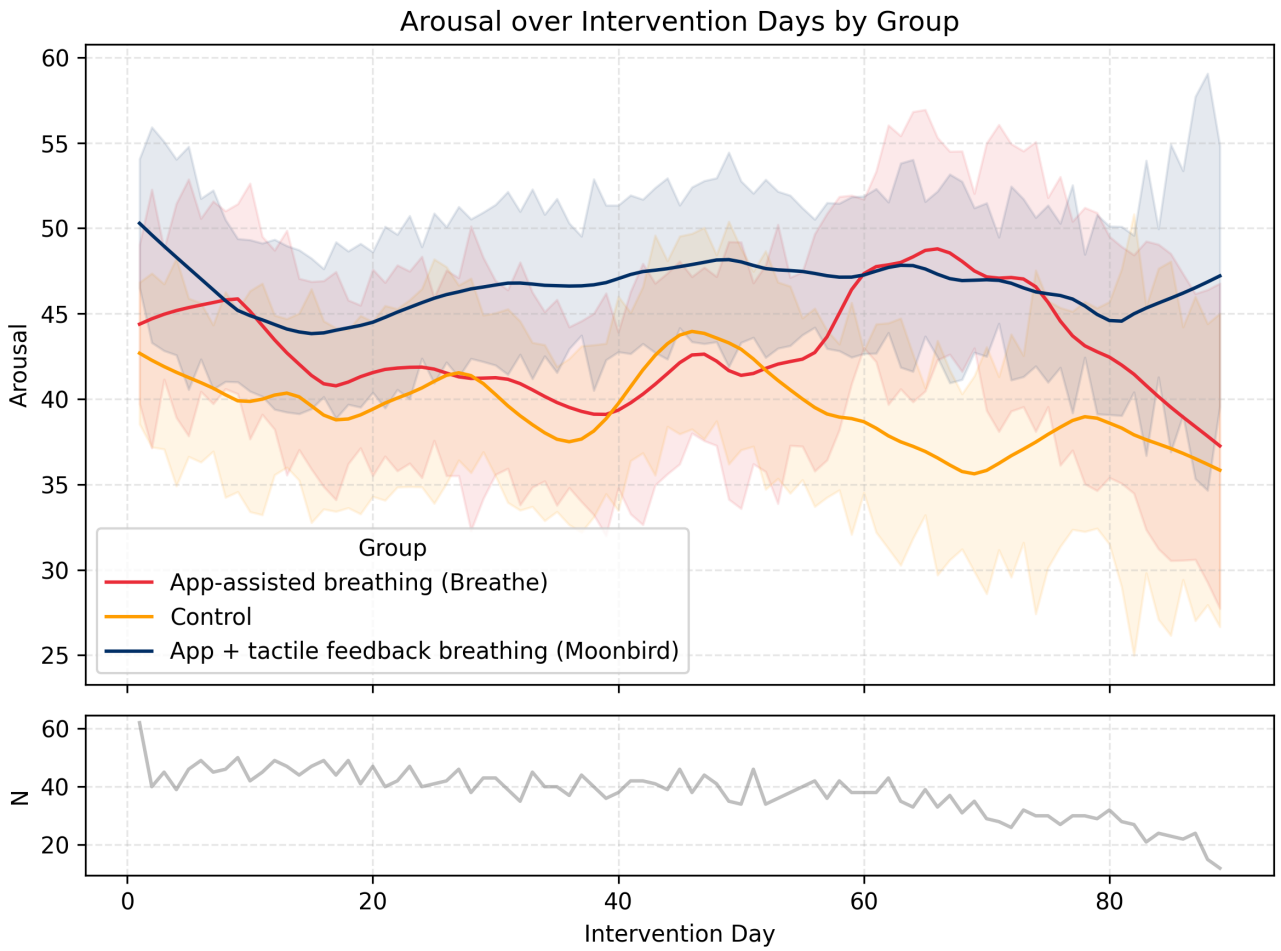
*Daily Arousal* levels and their evolution over time throughout the intervention period in the three experimental conditions. Shaded areas represent ± SEM. The lower graph (N) represents the number of participants who filled in the EMA survey on any given day.

*Daily Arousal.* There was no main effect of Group, F(2,3921)=0.99, p=0.37, and no overall change across time, F(1,3921)=0.02, p=0.90. The Group × Time interaction was also not significant, F(2,3921)=2.98, p=0.051. The trajectory of *Daily Arousal* across the intervention period is illustrated in Figure 1.

**Figure 2:**
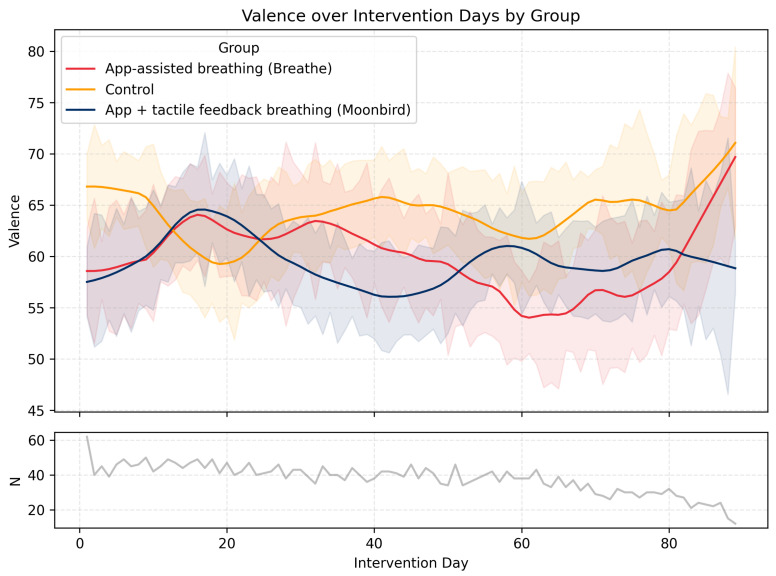
Daily *Valence* levels and their evolution over time throughout the intervention period in the three experimental conditions. Shaded areas represent ± SEM. The lower graph (N) represents the number of participants who filled in the EMA survey on any given day.

*Daily Valence.* There was no main effect of Group, F(2,3921)=0.37, p=0.69, and no overall change across time, F(1,3921)=0.96, p=0.33. The Group × Time interaction was likewise non-significant, F(2,3921)=1.80, p=0.17. The trajectory of *Daily Valence* across the intervention period is illustrated in Figure 2.

**Figure 3:**
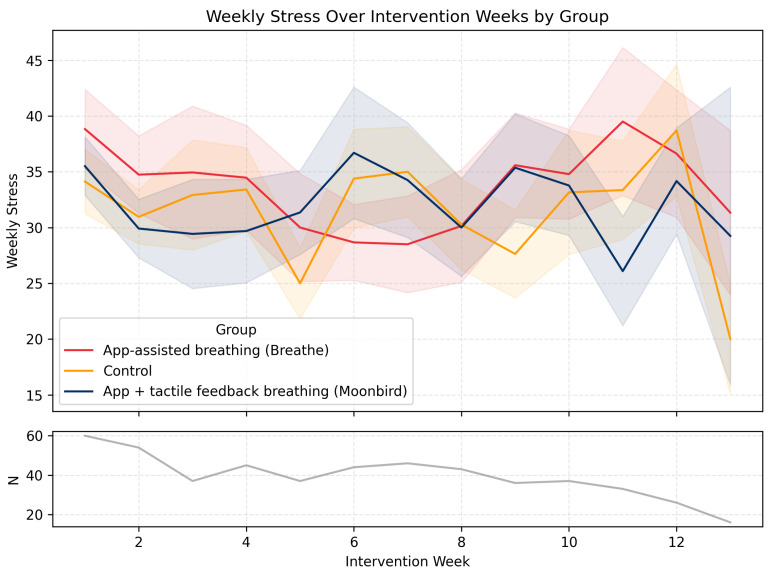
*Weekly Stress* levels and their evolution over time throughout the intervention period in the three experimental conditions. Shaded areas represent ± SEM. The lower graph (N) represents the number of participants who filled in the EMA survey on any given week.

*Weekly Stress.* Again, there was no main effect of Group, F(2,721)=0.16, p=0.85. However, weekly stress declined reliably over time, F(1,721)=71.22, p<0.001. The Group × Time interaction was not significant, F(2,721)=1.52, p=0.22. The trajectory of *Weekly Stress* across the intervention period is illustrated in Figure 3.

**Figure 4:**
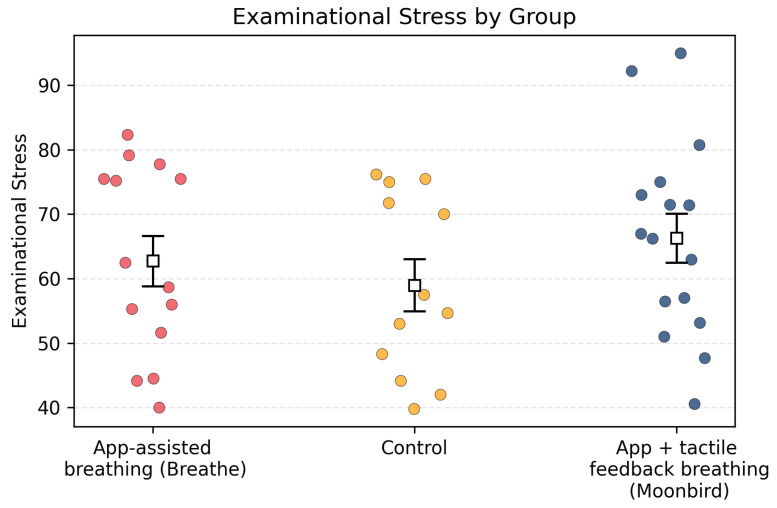
*Examinational Stress* levels in the three experimental conditions. Error bars represent ± SEM.

*Examinational Stress.* To test **H3** (lower examinational stress in breathing groups) at the group level, a one-way between-subjects ANOVA compared mean examination-stress scores across the three conditions (see Figure 4). The analysis revealed no significant group differences, F(2,43)=0.09, p=0.91. Thus, participants’ reported examination stress did not vary as a function of group assignment.

### 3.2. Within-Subjects Results

**H4** predicted that the effectiveness of breathing exercises would be moderated by baseline affect; that is, that individuals experiencing worse mood or higher arousal on the day of practice would show greater next-day improvements. To test this, a series of linear mixed-effects regression models clustered on participants were estimated to test whether performing breathing exercises at time t−1 affects valence and arousal at time *t* (models indexed with a), whether this relation is moderated by valence or arousal at time t−1 (models indexed with b), and whether this relation is further moderated by user experience in line with **H5** (models indexed with c). The full model results are presented in Table 2 and Table 3.

#### 3.2.1. Next-Day Arousal

**Figure 5:**
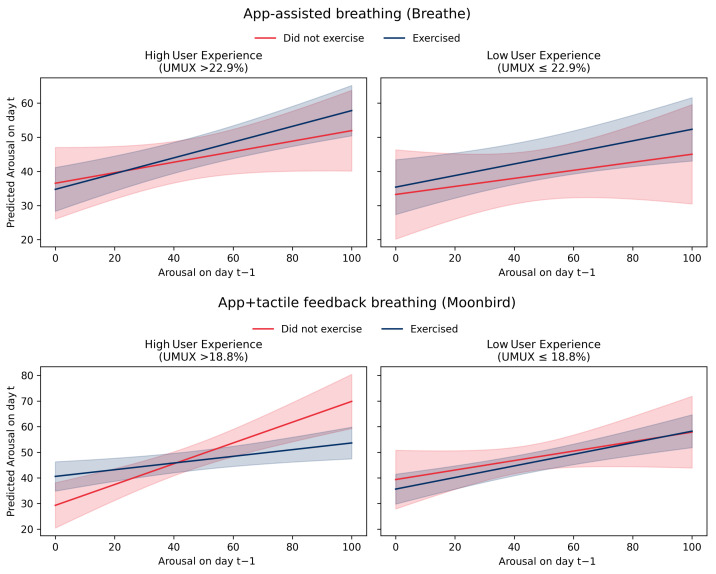
Predicted relation between *Arousal* on day t−1 and day *t* based on Model 2c (Table 2), visualizing the three-way interaction between *Arousal*_*t*−1_, *Adherence*, and *User Experience* in the app + haptic feedback group (Moonbird™). Each panel shows the simple slope of *Arousal*_*t*−1_ on *Arousal_t_* separately for days when the breathing exercise was performed versus not performed, and for participants with high versus low *User Experience* (split at the within-group median). Shaded areas are 95% CIs. A steeper negative slope for the adherence = yes/high user experience combination indicates that breathing exercise practice attenuated the carry-over of elevated arousal from one day to the next.

In partial support of **H4** and **H5**, the results revealed that performing breathing exercises had no main effect on arousal at time *t*, neither in the group which performed app-assisted breathing exercises (Breathe™) nor in the group that performed app + haptic device-assisted breathing exercises (Moonbird™). However, participants who experienced higher arousal at time t−1 and performed their breathing exercises reported significantly lower arousal at time *t*, but this was the case only in the app + haptic device-assisted breathing exercises (Moonbird™) group. Further analysis revealed that this effect was driven by participants with high user experience. A three-way interaction between adherence, ${\mathrm{arousal}}_{t-1}$ and user experience was found. The calming effect of a breathing exercise with Moonbird™ depended jointly on (i) how aroused the participant felt before practice and (ii) how positively they evaluated the device. Among high-user experience users, a session substantially weakened the day-to-day carry-over of arousal; among low-user experience users, the carry-over was virtually identical whether or not they practiced. This relation is visualized in Figure 5.

#### 3.2.2. Next-Day Valence

**Figure 6:**
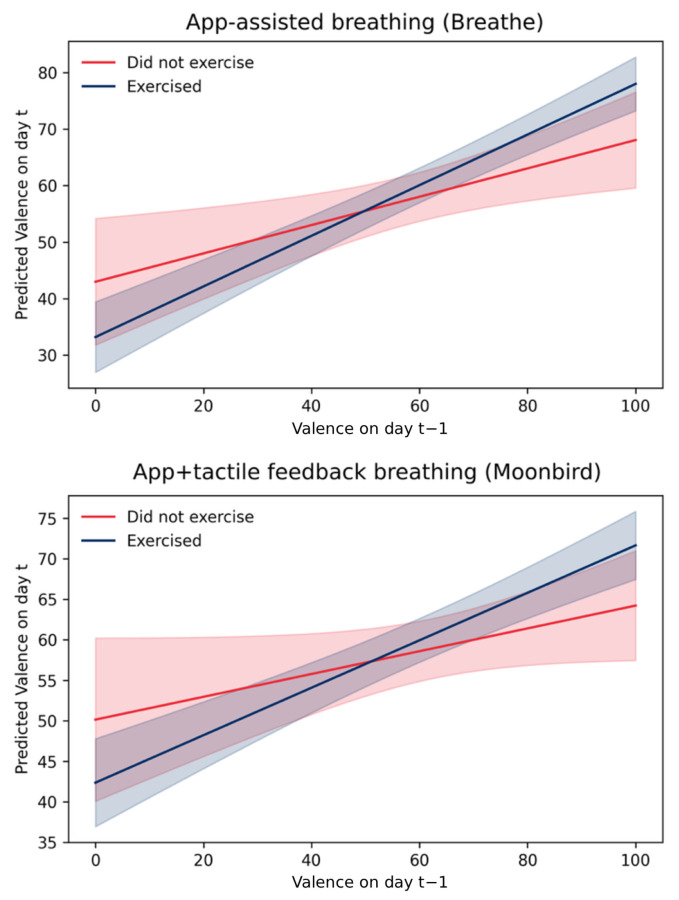
Predicted relation between *Valence* on day t−1 and day *t* based on Model 3b and Model 4b (Table 3), visualizing the two-way interaction between *Valence*_*t*−1_ and *Adherence* separately for the app-assisted (Breathe™) and app + haptic feedback (Moonbird™) groups. Each panel shows the simple slope of *Valence*_*t*−1_ on *Valence_t_* for days when the breathing exercise was performed versus not performed. Shaded areas are 95% CIs. A steeper positive slope for the adherence = yes condition indicates that breathing exercise practice strengthened the carry-over of positive valence from one day to the next.

In partial support of **H4**, the results revealed that performing breathing exercises had no main effect on valence at time *t*, neither in the group which performed app-assisted breathing exercises (Breathe™) nor in the group that performed app + haptic device-assisted breathing exercises (Moonbird™). However, participants who experienced higher valence at time t−1 and performed their breathing exercises reported significantly higher valence at time *t*, both in the app + haptic device-assisted breathing exercises (Moonbird™) group and in the app-assisted breathing exercises (Breathe™) group. In other words, the exercises did not lift mood in general, but strengthened an already positive mood trajectory. Further analysis of the three-way interaction revealed that this effect was not contingent on user experience, providing no support for **H5** in the valence domain. This relation is visualized in Figure 6.

#### 3.2.3. Weekly Stress

We further examined as an exploratory analysis whether **H4** extended to the weekly stress level—that is, whether weeks with greater adherence to breathing exercises predicted lower stress that same week, and whether this was moderated by prior stress levels or user experience (**H5)**. These models are presented in Table 4.

The results revealed that weekly stress levels were unaffected by breathing exercises, regardless of type, and that neither prior stress levels nor user experience moderated that lack of relation. These results do not support H4 at the within-person level for Weekly Stress.

#### 3.2.4. Examinational Stress

To further examine H3 at the within-person level, and to test whether H5 (UX moderation) extended to examinational stress outcomes, a series of exploratory models were estimated testing whether total adherence across the semester predicted examinational stress, and whether this was moderated by average weekly stress and user experience. The full model results are presented in Table 5.

**Figure 7:**
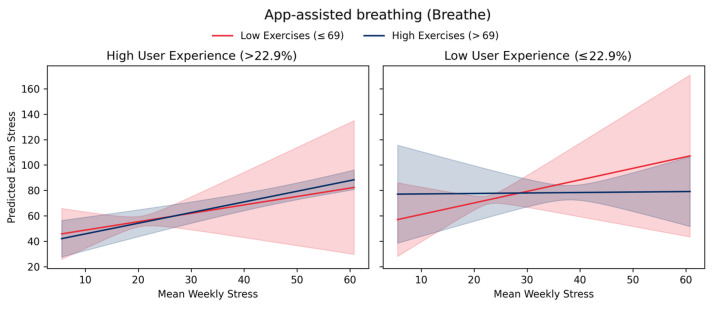

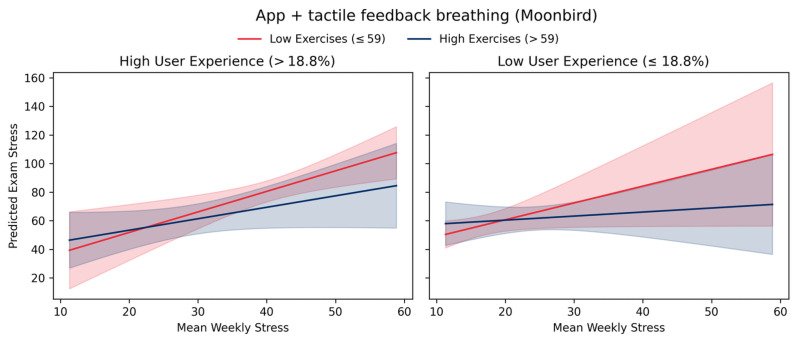
Predicted relation between average *Weekly Stress* throughout the semester and *Examinational Stress* based on Model 7c (Table 5) for the app-assisted group (Breathe™), visualizing the three-way interaction between *WeeklyStress_Total_*, *Adherence_Total_*, and *User Experience*. Each panel shows the simple slope of *WeeklyStress_Total_* on *Examinational Stress* separately for high versus low total adherence and high versus low *User Experience* (both split at the within-group median). Shaded areas are 95% CIs. The pattern indicates that higher weekly stress predicted greater examinational stress only among participants with low adherence or poor user experience; among highly adherent participants with positive user experience, this relationship was substantially attenuated.

The results revealed that performing breathing exercises had no main effect on Examinational Stress. However, both in the app + haptic device-assisted breathing exercises (Moonbird™) group and in the app-assisted breathing exercises (Breathe™) group, participants who experienced more weekly stress throughout the intervention and performed more breathing exercises (adherence), experienced greater reductions in examinational stress. Furthermore, in the app-assisted breathing exercises (Breathe™) a three-way interaction effect was found between adherence, weekly stress and user experience, indicating that the benefit of doing many breathing sessions only reduces examinational stress when the Breathe™ app feels subjectively good to use (see Figure 7), albeit the effect is relatively minimal. These results provide provisional evidence that breathing exercises could help with examinational stress, but only in those students who experience more stress, perform more exercises during the semester and have better user experience with their app/device.

## 4. Discussion

The present study investigated the impact of a technology-assisted, two-month breathing exercise intervention on daily affect, weekly stress, and examinational stress, comparing app-guided breathing exercises alone versus combined with a haptic biofeedback device (Moonbird™) against a no-intervention control group. Using ecological momentary assessment in participants’ natural environments, we examined both between-group differences in overall stress reduction and within-person dynamics to capture the real-world efficacy of these interventions. Overall, H1 (main effect of breathing on daily affect and weekly stress), H2 (augmentation by haptic feedback), and H3 (lower examinational stress in breathing groups) were not supported at the between-subjects level. H4 (moderation of next-day affect by baseline affective state) received partial support, with effects emerging for arousal in the Moonbird™ group and for valence in both active groups. H5 (moderation by user experience) was supported for arousal in the Moonbird™ group but not for valence or weekly stress.

It should further be noted that the between-subjects tests of H1, H2, and H3 were fully confirmatory, whereas the within-subjects moderation analyses testing H4 and H5—while pre-specified at the hypothesis level—involved interaction structures more complex than those originally anticipated, and the weekly stress and examinational stress within-subjects models were conducted as exploratory analyses. Critically, the interaction effects involving user experience and adherence reported below should be interpreted as preliminary and hypothesis-generating rather than confirmatory, and replicated in adequately powered pre-registered studies before informing applied recommendations.

Contrary to our first hypothesis, neither breathing intervention group demonstrated improvements in daily affect or weekly stress compared to the control group. Between-subjects analyses revealed no significant main effects of group assignment on daily arousal, daily valence, or weekly stress levels. Notably, weekly stress declined significantly over time across all groups, but this improvement was not attributable to the breathing interventions, as evidenced by the non-significant Group × Time interaction. These null findings appear to contrast with recent meta-analytic evidence: Fincham et al. (2023) reported a small-to-medium pooled effect on self-reported stress (g=−0.35, 95% CI −0.55 to −0.14) across 12 randomized controlled trials, suggesting that breathing interventions typically produce modest but meaningful reductions. However, our findings align with emerging evidence from ecological momentary intervention studies that have similarly reported null quantitative effects despite qualitative user reports of benefit. Perreau et al. (2023), for instance, found no pre-post changes on standardized stress measures following a smartphone-based breathing intervention, despite interview data revealing immediate somatic and cognitive changes after exercises—suggesting that breathing benefits may be transient and confined to practice sessions rather than producing sustained daily improvements.

Several methodological factors may explain the discrepancy between our null findings and meta-analytic evidence, though we acknowledge these explanations are post hoc and would require prospective validation through pre-registered studies with adequate power. First, ecological momentary assessment captures real-time fluctuations in affect and stress, which may be more sensitive to momentary variability but less sensitive to the modest average effects typically detected in pre-post designs. Second, our healthy, predominantly young adult sample may have experienced ceiling effects, as breathing interventions have shown larger effect sizes in clinical populations with elevated baseline distress (Toussaint et al., 2021). Third, the general decline in weekly stress across all groups suggests potential secular trends or study participation effects that may have masked intervention-specific benefits—a phenomenon increasingly recognized in digital health research where control groups often show unexpected improvements (Smits et al., 2022).

Our findings contribute to a growing body of literature highlighting the complexity of translating laboratory-demonstrated breathing benefits to real-world contexts. The modest pooled effect size from meta-analyses suggests that individual studies, particularly those using intensive longitudinal designs like EMA, may be underpowered to detect these small-to-moderate effects in healthy populations, pointing to the need for larger samples or more targeted recruitment of distressed individuals.

Our second hypothesis, that the haptic breathing device would augment the effects of app-guided breathing exercises, received mixed support. Between-subjects analyses showed no differential benefits of the app + device condition over the app-only condition. However, within-subjects analyses revealed a moderation effect: participants in the Moonbird™ group who experienced higher arousal at time t−1 and performed breathing exercises reported significantly lower arousal at time *t*, contingent on positive user experience ratings. These findings align with recent controlled trials demonstrating conditional rather than universal effectiveness of haptic biofeedback devices. Farrall et al. (2023) compared a handheld shape-changing biofeedback device (PAWS) to an audio-only control during guided breathing exercises and found significant reductions in both physiological and self-reported anxiety in the haptic condition, with benefits most pronounced among participants who engaged meaningfully with the device—supporting our finding that user experience moderates technological benefits. Hadi and Valenzuela (2020) similarly demonstrated that vibrational haptic cues improve task performance and perceived social presence, but with small effect sizes highly dependent on user engagement and acceptance. It should be noted, however, that the Moonbird™ condition differed from the app-only condition not only in the presence of haptic feedback but also in the availability of real-time physiological data and post-session metric summaries. The within-subjects effects observed in the haptic group—and, in particular, the moderation by user experience—may therefore have been driven in part or in whole by this additional biofeedback information rather than by haptic guidance per se, and this alternative explanation cannot be ruled out on the basis of the present data.

Our third hypothesis, that breathing exercise groups would report lower examinational stress compared to controls, was not supported by the between-subjects analysis. However, the within-subjects analyses revealed a more complex pattern: participants in both breathing groups who experienced higher weekly stress throughout the intervention and maintained higher adherence reported greater reductions in examinational stress. This effect was further moderated by user experience in the app-only group, where the benefits of frequent breathing practice during stressful periods were contingent on positive app evaluations. These results align with recent evidence suggesting that breathing interventions may be most effective when implemented during periods of elevated stress rather than as general wellness practices. Buttazzoni et al. (2021) found in a systematic review and meta-analysis of smartphone-based interventions for internalizing disorders in youth that effectiveness was consistently moderated by usability and engagement factors, with poorly designed or low-engagement interventions showing minimal benefits even when based on evidence-based techniques.

Our fourth hypothesis received partial support. While breathing exercises did not show universal next-day predictive effects, we found evidence for the hypothesized moderation by baseline affective state. Specifically, participants who experienced higher arousal and performed breathing exercises reported lower arousal the following day, but only in the Moonbird™ group and only among those with positive user experience. Conversely, breathing exercises appeared to strengthen already positive mood trajectories in both intervention groups. These findings offer preliminary evidence for differential mechanisms of breathing interventions across affective domains. The arousal pattern—where benefits emerged primarily when starting from elevated states—aligns with regulatory theories suggesting that breathing exercises function as corrective tools most beneficial for individuals experiencing dysregulation, consistent with Toussaint et al. (2021), who found the largest physiological relaxation effects among participants with initially elevated stress markers. The contrasting valence pattern suggests a different mechanism: rather than correcting negative states, breathing exercises may maintain or enhance positive emotional experiences, aligning with emerging research on positive emotion regulation. Our within-subjects approach to examining next-day effects offers a complementary perspective to the predominantly pre-post designs in this literature, and the temporal specificity of our findings supports recent calls for intensive longitudinal designs to capture the person-specific processes underlying intervention effects.

### 4.1. Limitations

Despite the methodological rigor of employing ecological momentary assessment in a randomized controlled trial, several limitations warrant consideration for interpreting these findings and designing future research. The study’s sample size of 62 participants, while adequate for detecting large effect sizes, may have been underpowered to detect the modest between-subjects effects typically observed in breathing intervention research. This limitation is particularly relevant for the examinational stress analyses, which included only 45 student participants, further reducing statistical power. However, this limitation is partially counteracted by the EMA design, which yielded a total of 667 observations for the analyses of day-by-day changes in valence and arousal. A sensitivity analysis conducted in G*Power (v. 3.1) (one-way ANOVA, α=0.05, power =0.80) indicated that the full sample of 62 participants provided adequate power to detect only large between-group effects (f=0.40). For the examinational stress analyses, which were based on the 45 student participants, the minimum detectable effect was even larger (f=0.48). Given that meta-analytic evidence for breathing interventions suggests pooled effect sizes in the small-to-medium range (g≈0.35; (Fincham et al., 2023)), the present study was underpowered to detect between-subjects effects of the magnitude typically reported in this literature. The null between-subjects findings should therefore be interpreted cautiously as inconclusive rather than as evidence of absence.

The sample consisted predominantly of young, healthy adults who were excluded if they had anxiety disorders or regularly practiced breathing exercises. This selection bias limits generalizability to people experiencing chronic stress who might show greater responsiveness to breathing interventions, as suggested by research showing larger effect sizes in distressed versus healthy populations (Toussaint et al., 2021). Additionally, the exclusion of individuals already practicing breathing exercises, while methodologically sound for maintaining control group integrity, may have excluded those most likely to benefit from such interventions. The gender imbalance also raises questions about generalizability to male populations, particularly given potential gender differences in technology acceptance and stress coping strategies (Graves et al., 2021).

A further limitation concerns the non-equivalence of the two app interfaces used across active conditions. The Breathe™ app provided visual pacing, voice narration, and ambient soundscapes, while the Moonbird™ app offered a qualitatively different interface featuring real-time physiological monitoring (HR, HRV, and coherence) and post-session metric summaries. These differences mean that the two active conditions were not matched on app-level features, and any differential effects observed between them cannot be attributed exclusively to the presence or absence of haptic feedback. In particular, the continuous availability of biofeedback data in the Moonbird™ group may have independently influenced engagement, self-awareness, or perceived intervention value—all of which could plausibly contribute to the user experience moderation effects observed in that group. This confound is most consequential for the interpretation of between-group comparisons, where differences could reflect app characteristics rather than haptic guidance per se. It is less critical for the within-subjects analyses, which concern day-to-day dynamics within each group rather than differences between them, but it does limit the specificity of any conclusions about what aspect of the haptic condition drove the observed effects. Future studies seeking to isolate the contribution of haptic breathing guidance should hold the app interface constant across conditions, varying only the haptic component, ideally using a version of the same app with haptic cues enabled versus disabled.

While adherence was measured daily through self-report, this approach is subject to several well-recognized limitations. Specifically, self-reported adherence may be affected by recall bias, given that participants were asked whether they performed their exercise the *previous* day, and by social desirability bias, as participants aware of the study’s goals may have over-reported compliance. The single-item format further limits insight into the quality or intensity of each session. Objective usage data were not made available to the research team, due to personal data protection laws, and could therefore not be used as an objective adherence measure. Future studies involving commercial breathing applications or devices should establish data-sharing agreements with developers prior to data collection, enabling objective verification of session completion and exploration of dose-response relationships. The absence of physiological monitoring during exercises (e.g., heart rate variability, breathing rate) further represents a missed opportunity to verify intervention fidelity and delivery quality (Lange et al., 2011).

The intervention prescribed a single 6 min daily box-breathing session. While the dose-response literature suggests that five minutes represents a practical minimum threshold for physiological effects—with no additional benefit from extending individual sessions beyond this point (Bentley et al., 2023; You et al., 2021)—and while Balban et al. (2023) demonstrated significant cumulative mood benefits from daily five-minute sessions over 28 days, it should be noted that most of this evidence is based on physiological outcomes such as HRV rather than self-reported psychological stress. The possibility that longer individual sessions or higher cumulative weekly doses might be required to produce detectable between-subjects effects on subjective stress in healthy populations cannot be excluded, and future studies should systematically vary session duration to establish dose-response curves for psychological outcomes.

The intervention duration varied between 61 and 95 days across participants (M=86.9±9.4 days) as a consequence of staggered recruitment, with all participants concluding on the same fixed end date. This variability represents a potential confounder: participants recruited earlier received somewhat longer exposure to the intervention, which may have influenced the effectiveness of the intervention. Future studies should either fix intervention duration across participants or statistically control for exposure length.

The significant decline in weekly stress across all groups, including controls, suggests potential contamination or secular trends that may have masked intervention effects. This phenomenon has been increasingly recognized in digital health research, where control groups often show unexpected improvements (Waters et al., 2012). A related concern is that the no-intervention control design does not control for expectancy effects: participants assigned to active conditions may have improved partly due to the belief that they were receiving an effective intervention rather than due to the breathing exercises per se. Future studies should therefore consider active control conditions that equate for expectancy, such as placebo breathing exercises using non-therapeutic rhythms, or comparison against an established relaxation technique.

The reliance on self-report measures, while appropriate for subjective experiences like affect and stress, introduces potential response bias and limits objective assessment of intervention effects. The adapted Self-Assessment Manikin for daily affect measurement, while validated, may lack sensitivity to detect subtle changes in emotional states. Additionally, the single-item adherence measure provides limited information about the quality or intensity of breathing practice. Recent research has emphasized the importance of multi-modal assessment in health intervention studies, including physiological markers of stress (e.g., cortisol, heart rate variability) and more comprehensive adherence assessments (Strehli et al., 2021). Future research should incorporate these measures to complement self-report data.

Despite these limitations, the study’s ecological validity, longitudinal design, and focus on real-world implementation provide valuable insights into the practical effectiveness of technology-assisted breathing interventions. The findings highlight the importance of individual differences, particularly user experience and baseline affective states, in determining intervention success, offering potential directions for research on human “computer” interaction, investigating the user experience and how to improve it, for example by personalizing the interface.

### 4.2. Conclusions

This study provides modest, partially exploratory evidence for the effectiveness of technology-assisted breathing interventions in real-world settings, revealing that benefits are conditional rather than universal. In particular, findings involving moderation by user experience and adherence emerge from exploratory interaction models and should be interpreted cautiously pending replication. The haptic biofeedback device showed promise for reducing arousal among users who evaluated it positively and were experiencing elevated stress, supporting recent controlled trials demonstrating conditional effectiveness of haptic feedback technologies. These findings replicate the critical importance of user experience as a moderator of digital health intervention effectiveness, lending further quantitative support for the growing emphasis on user-centered design in this field. Future research should focus on developing adaptive systems that can identify optimal intervention timing (when arousal is high) and modality based on individual user profiles, while addressing the methodological limitations identified here through larger, more diverse samples and multi-modal outcome measures.

## Figures and Tables

**Table 1:** Descriptive statistics of the study variables averaged over the entire intervention period. Error values are ±SD.

Variable	App + Haptic Assisted Breathing (Moonbird™)	App-Assisted Breathing (Breathe™)	Control
*Arousal*	46.79±7.74	42.09±13.02	40.40±12.37
	(n=20)	(n=20)	(n=21)
*Valence*	59.47±7.91	61.24±10.97	63.57±10.00
	(n=20)	(n=20)	(n=21)
*Weekly Stress*	26.88±13.33	25.50±15.89	26.97±12.04
	(n=20)	(n=20)	(n=20)
*Examinational Stress*	50.28±29.03	46.69±22.56	48.83±16.06
	(n=19)	(n=15)	(n=12)
*Performed exercises*	57.45±20.14 ^1^	51.30±25.40 ^1^	–
*(Adherence)*	(n=20)	(n=20)	

^1^ The difference in adherence between the Moonbird and Breathe groups was not statistically significant, *t*(36) = 0.85, *p* = 0.40.

**Table 2:** Results of the regression analyses testing whether performing breathing exercises the day before affects today’s arousal and whether this effect is moderated by yesterday’s arousal and user experience. Values in parentheses are standard errors of the coefficients; *r* values (computed as $z/\sqrt{N}$) are reported in square brackets for significant predictors as effect size indices. Variable names are shown in italics, significant coefficients are shown in bold. *—p<0.05, **—p<0.01.

Outcome	*Arousal_t_*					
Group	App-Assisted Breathing (Breathe™)			App + Haptic Feedback Breathing (Moonbird™)		
Predictor (n)	Model 1a	Model 1b	Model 1c	Model 2a	Model 2b	Model 2c
Intercept	41.59 (6.27) **	44.34 (7.32) **	43.91 (9.05) **	40.20 (5.04) **	33.92 (5.96) **	50.38 (9.87) **
*Arousal*_*t*−1_	0.20 (0.05) ** [*r* = 0.16]	0.15 (0.09)	0.11 (0.14)	0.21 (0.04)** [*r* = 0.18]	0.36 (0.08) ** [*r* = 0.15]	−0.00 (0.20)
*Valence*_*t*−1_	−0.20 (0.05) ** [*r* = −0.16]	−0.20 (0.05) ** [*r* = −0.16]	−0.20 (0.05) ** [*r* = −0.16]	−0.04 (0.04)	−0.05 (0.04)	−0.04 (0.04)
*Adherence_t_*	2.81 (2.21)	−0.24 (4.77)	3.46 (7.57)	−1.46 (1.74)	6.47 (4.35)	−16.58 (9.79)
*User Experience*	0.11 (0.12)	0.11 (0.11)	0.13 (0.23)	0.02 (0.14)	0.02 (0.14)	−0.69 (0.37)
*Adherence_t_* × *Arousal*_*t*−1_		0.07 (0.09)	0.04 (0.15)		−0.18 (0.09) * [*r* = −0.07]	0.31 (0.21)
*Adherence_t_* × *User Experience*			0.00 (0.00)			1.03 (0.39) ** [*r* = 0.09]
*Arousal*_*t*−1_ × *User Experience*			−0.16 (0.23)			0.02 (0.01) * [*r* = 0.07]
*Arousal*_*t*−1_ × *Adherence_t_* × *User Experience*			0.00 (0.01)			−0.02 (0.01) ** [*r* = −0.07]
Observations	**667**	**667**	**667**	**861**	**861**	**861**
Marginal/Conditional $R^{2}$	**0.14/0.29**	**0.14/0.29**	**0.14/0.29**	**0.05/0.16**	**0.06/0.16**	**0.06/0.17**
ΔMarginal $R^{2}$	**—**	**0.00**	**0.00**	**—**	**+0.01**	**0.00**
Cohen’s $f^{2}$ (marginal)	**0.163**	**0.163**	**0.163**	**0.053**	**0.064**	**0.064**
Wald $\chi^{2}$	92.16 **	92.77 **	95.35 **	42.63 **	46.78 **	54.97 **

**Table 3:** Results of the regression analyses testing whether performing breathing exercises the day before affects today’s valence and whether this effect is moderated by yesterday’s valence and user experience. Values in parentheses are standard errors of the coefficients; *r* values (computed as $z/\sqrt{N}$) are reported in square brackets for significant predictors as effect size indices. Variable names are shown in italics, significant coefficients are shown in bold. *—p<0.05, **—p<0.01.

Outcome	*Valence_t_*					
Group	App-Assisted Breathing (Breathe™)			App + Haptic Feedback Breathing (Moonbird™)		
Predictor (n)	Model 3a	Model 3b	Model 3c	Model 4a	Model 4b	Model 4c
Intercept	34.04 (5.17) **	44.16 (6.86) **	53.51 (9.36) **	45.53 (4.58) **	52.68 (6.04) **	53.40 (10.45) **
*Valence*_*t*−1_	0.44 (0.05) ** [*r* = 0.34]	0.26 (0.09) ** [*r* = 0.11]	0.10 (0.14)	0.26 (0.04) ** [*r* = 0.22]	0.14 (0.08)	0.13 (0.16)
*Arousal*_*t*−1_	0.01 (0.04)	0.01 (0.04)	0.00 (0.04)	−0.04 (0.04)	−0.04 (0.04)	−0.03 (0.04)
*Adherence_t_*	1.39 (1.93)	−10.36 (5.61)	−13.51 (8.92)	1.57 (1.64)	−7.77 (5.38)	−17.20 (11.21)
*User Experience*	−0.09 (0.08)	−0.08 (0.08)	−0.37 (0.23)	−0.05 (0.12)	−0.05 (0.12)	−0.09 (0.37)
*Adherence_t_* × *Valence*_*t*−1_		0.20 (0.09) * [*r* = 0.09]	0.26 (0.15)		0.16 (0.08) * [*r* = 0.07]	0.29 (0.18)
*Adherence_t_* × *User Experience*			0.07 (0.25)			0.42 (0.42)
*Valence*_*t*−1_ × *User Experience*			0.01 (0.00)			0.00 (0.00)
*Valence*_*t*−1_ × *Adherence_t_* × *User Experience*			−0.00 (0.00)			−0.01 (0.01)
Observations	**667**	**667**	**667**	**861**	**861**	**861**
Marginal/Conditional $R^{2}$	**0.22/0.31**	**0.23/0.31**	**0.12/0.32**	**0.08/0.17**	**0.09/0.18**	**0.10/0.18**
ΔMarginal $R^{2}$	**—**	**+0.01**	**−0.11**	**—**	**+0.01**	**+0.01**
Cohen’s $f^{2}$ (marginal)	**0.282**	**0.299**	**0.136**	**0.087**	**0.099**	**0.111**
Wald $\chi^{2}$	147.05 **	153.08 **	160.30 **	70.71 **	74.31 **	77.58 **

**Table 4:** Results of the regression analyses testing whether performing breathing exercises during the week affects weekly stress and whether this effect is moderated by the previous week’s stress and user experience. Values in parentheses are standard errors of the coefficients; *r* values (computed as $z/\sqrt{N}$) are reported in square brackets for significant predictors as effect size indices. Variable names are shown in italics, significant coefficients are shown in bold. *—p<0.05, **—p<0.01.

Outcome	*WeeklyStress_t_*					
Group	App-Assisted Breathing (Breathe™)			App + Haptic Feedback Breathing (Moonbird™)		
Predictor (n)	Model 5a	Model 5b	Model 5c	Model 6a	Model 6b	Model 6c
Intercept	44.58 (9.17) **	41.77 (9.98) **	39.51 (10.63) **	22.07 (8.07) **	26.88 (9.10) **	26.21 (11.81) **
*Valence_t_*	−0.37 (0.09) ** [*r* = −0.32]	−0.37 (0.09) ** [*r* = −0.32]	−0.38 (0.09) ** [*r* = −0.33]	−0.30 (0.07) ** [*r* = −0.31]	−0.31 (0.07) ** [*r* = −0.32]	−0.31 (0.07) ** [*r* = −0.32]
*Arousal_t_*	0.07 (0.08)	0.07 (0.08)	0.07 (0.09)	0.28 (0.07) ** [*r* = 0.29]	0.27 (0.07) ** [*r* = 0.28]	0.28 (0.07) ** [*r* = 0.29]
*WeeklyStress*_*t*−1_	−0.13 (0.08)	0.24 (0.16)	0.26 (0.20)	0.44 (0.07) ** [*r* = 0.46]	0.31 (0.13) * [*r* = 0.17]	0.26 (0.27)
*Adherence_t_*	0.83 (0.50)	1.35 (0.90)	1.88 (1.56)	−0.18 (0.47)	−1.09 (0.94)	−1.59 (1.64)
*User Experience*	−3.93 (4.76)	−3.99 (4.70)	1.23 (11.74)	2.88 (3.33)	3.07 (3.35)	6.09 (11.37)
*Adherence_t_* × *WeeklyStress*_*t*−1_		−0.02 (0.02)	−0.02 (0.03)		0.03 (0.02)	0.06 (0.05)
*Adherence_t_* × *User Experience*			−0.65 (2.01)			0.33 (2.05)
*WeeklyStress*_*t*−1_ × *User Experience*			0.02 (0.32)			0.03 (0.30)
*WeeklyStress*_*t*−1_ × *Adherence_t_* × *User Experience*			−0.02 (0.06)			−0.04 (0.05)
Observations	**166**	**166**	**166**	**187**	**187**	**187**
Marginal/Conditional $R^{2}$	**0.33/0.71**	**0.34/0.71**	**0.35/0.71**	**0.57/0.75**	**0.57/0.75**	**0.57/0.76**
ΔMarginal $R^{2}$	**—**	**+0.01**	**+0.01**	**—**	**0.00**	**0.00**
Cohen’s $f^{2}$ (marginal)	**0.493**	**0.515**	**0.538**	**1.326**	**1.326**	**1.326**
Wald $\chi^{2}$	73.96 **	74.93 **	77.46 **	123.51 **	126.45 **	129.90 **

**Table 5:** Results of the regression analyses testing whether performing breathing exercises during the semester affects examinational stress and whether this effect is moderated by weekly stress and user experience. Values in parentheses are standard errors of the coefficients; *r* values (computed as $t/\sqrt{t^{2}+df}$) are reported in square brackets for significant predictors as effect size indices. Variable names are shown in italics, significant coefficients are shown in bold. *—p<0.05, **—p<0.01.

Outcome	*Examinational Stress*					
Group	App-Assisted Breathing (Breathe™)			App + Haptic Feedback Breathing (Moonbird™)		
Predictor (n)	Model 7a	Model 7b	Model 7c	Model 8a	Model 8b	Model 8c
Intercept	77.63 (62.98)	−1.14 (70.71)	−158.7 (73.2) *	28.60 (69.42)	26.16 (54.15)	36.13 (492.0)
*Valence_Total_*	−0.18 (0.72)	0.57 (0.82)	0.19 (1.14)	0.37 (0.44)	0.38 (0.41)	0.38 (0.38)
*Arousal_Total_*	−0.21 (0.48)	0.03 (0.45)	−0.10 (0.70)	0.09 (0.38)	0.22 (0.44)	0.44 (0.51)
*WeeklyStress_Total_*	0.23 (0.37)	3.12 (1.31) ** [*r* = 0.31]	11.20 (3.83) ** [*r* = 0.38]	0.88 (0.25) ** [*r* = 0.45]	2.40 (0.82) ** [*r* = 0.39]	4.38 (18.77)
*Adherence_Total_*	0.29 (0.18)	0.77 (0.15) ** [*r* = 0.57]	5.79 (1.92) ** [*r* = 0.39]	−0.19 (0.21)	0.43 (0.37)	1.71 (8.05)
*User Experience*	−0.69 (0.78)	−1.45 (0.74) * [*r* = 0.26]	7.41 (2.49) ** [*r* = 0.38]	−0.16 (2.47)	−1.87 (2.39)	−3.07 (20.21)
*Adherence* × *WeeklyStress*		−0.04 (0.01) ** [*r* = 0.48]	−0.20 (0.07) ** [*r* = 0.37]		−0.02 (0.01) * [*r* = 0.28]	−0.11 (0.31)
*Adherence* × *User Experience*			−0.22 (0.08) ** [*r* = 0.36]			−0.05 (0.33)
*WeeklyStress* × *User Experience*			−0.40 (0.17) * [*r* = 0.31]			−0.07 (0.73)
*WeeklyStress* × *Adherence* × *User Experience*			0.01 (0.00) * [*r* = 0.27]			0.00 (0.01)
Observations	**61**	**61**	**61**	**56**	**56**	**56**
$R^{2}$	**0.48**	**0.58**	**0.64**	**0.43**	**0.51**	**0.52**
Δ $R^{2}$	**—**	**+0.10**	**+0.06**	**—**	**+0.08**	**+0.01**
Cohen’s $f^{2}$	**0.923**	**1.381**	**1.778**	**0.754**	**1.041**	**1.083**
F	F(4, 56) = 7.58 **	F(6, 54) = 15.77 **	F(9, 51) = 10.07 **	F(5, 50) = 6.54 **	F(6, 49) = 7.99 **	F(9, 46) = 11.86 **

## Data Availability

The data presented in this study are available on request from the corresponding author.

## Appendix A. Statistical Analysis

To test whether conditions differed in the daily valence and arousal levels and the weekly stress levels, a repeated-measures ANOVA was implemented as a linear mixed-effects model, the modern equivalent of the classical mixed-factor ANOVA but better suited to unequally spaced and incomplete diary data (Mundo et al., 2022). For each dependent variable—daily arousal, daily valence, and weekly stress—we specified Group (Moonbird™/Breathe™/Control) as the between-subjects factor, and Time (day index for the daily outcomes, week index for weekly stress) as the within-subjects factor. A random intercept for Participant absorbed the correlation introduced by repeated observations within the same individual. Models were fitted in Python 3.13 with statsmodels 0.15 (ML, LBFGS optimiser). Fixed-effect significance was assessed with joint Wald tests on the relevant coefficient block and converted to an *F*-statistic ($F=\chi^{2}/df$). This analysis addressed the between-subjects question: Do the app-assisted breathing exercises group (Breathe™), app + haptic feedback-assisted breathing exercises group (Moonbird™) and control group differ in their average level and temporal trajectory of valence, arousal and weekly stress across the study period? Additionally, in order to answer the question whether the three conditions differed in their levels of examinational stress, for each participant we averaged all completed Exam-Stress questionnaires and subjected these means to a one-way between-subjects ANOVA with Group as the sole factor (Python 3.13, statsmodels 0.15; ordinary least-squares fit, Type-II sums of squares).

To test whether completing a breathing exercise on one day predicted the next-day mood, we analyzed the intensive-longitudinal diary data with linear mixed effects models (Python 3.13, statsmodels 0.15). For each participant, we first created 1 day lags of self-reported valence and arousal and retained only true consecutive-day pairs (i.e., we only analyzed situations where participants provided two survey answers on two consecutive days). Three sets of two models were fitted separately for the Moonbird™ group and the Breathe™ group.

The main effect models asked: Do people who perform breathing exercises on day *n* feel differently on day n+1?

(A1) $${\mathrm{Outcome}}_{t}=\beta_{0}+\beta_{1}\cdot {\mathrm{Adherence}}_{t}+\beta_{2}\cdot {\mathrm{Outcome}}_{t-1}+\beta_{3}\cdot {\mathrm{CrossLag}}_{t-1}+\beta_{4}\cdot {\mathrm{UserExperience}}_{i}+u_{participant}+\varepsilon$$

where the CrossLag term was ${\mathrm{Valence}}_{t-1}$ when arousal was the outcome and vice versa.

The interaction models asked: Is the next day effect of breathing exercises moderated by how the person felt on the practice day and their user experience?

(A2) $$\begin{array}{cc} {\mathrm{Outcome}}_{t}= & \beta_{0}+\beta_{1}\cdot {\mathrm{Outcome}}_{t-1}+\beta_{2}\cdot {\mathrm{Adherence}}_{t}+\beta_{3}\cdot {\mathrm{UserExperience}}_{i}\\ \; & \;+\beta_{4}\cdot \left({\mathrm{Outcome}}_{t-1}\times {\mathrm{Adherence}}_{t}\right)\\ \; & \;+\beta_{5}\cdot \left({\mathrm{Outcome}}_{t-1}\times {\mathrm{UserExperience}}_{i}\right)\\ \; & \;+\beta_{6}\cdot \left({\mathrm{Adherence}}_{t}\times {\mathrm{UserExperience}}_{i}\right)\\ \; & \;+\beta_{7}\cdot \left({\mathrm{Outcome}}_{t-1}\times {\mathrm{Adherence}}_{t}\times {\mathrm{UserExperience}}_{i}\right)\\ \; & \;+\beta_{8}\cdot {\mathrm{CrossLag}}_{t-1}+u_{participant}+\varepsilon \end{array}$$

This strategy answers (i) whether same day practice predicts improved mood the following day after controlling for autocorrelation and (ii) whether that effect depends on the individual’s mood at the time of practice. Note that Adherence was indexed with *t* and not t−1 because the question posed to participants about whether they performed their breathing exercise asked about the previous day. As such, adherence values at *t* described what happened the day before (t−1).

To assess whether the change in weekly stress from one week to the next is shaped by the amount of breathing exercises performed during that week, we analyzed the intensive EMA data at the week grain with linear mixed-effects regression in Python 3.13 (statsmodels 0.15, LBFGS optimizer). TotalExercises indicates the amount of breathing exercises performed during the week leading up to the weekly stress survey; WeeklyArousal and WeeklyValence indicate mean of the daily arousal and valence ratings during that week.

The main effect models asked: Do people who perform more breathing exercises throughout the week experience different stress levels at the end of that week?

(A3) $$\begin{array}{c} {\mathrm{WeeklyStress}}_{t}=\beta_{0}+\beta_{1}\cdot {\mathrm{WeeklyStress}}_{t-1}+\beta_{2}\cdot {\mathrm{WeeklyExercises}}_{t}+\beta_{3}\cdot \mathrm{UserExperience}\\ +\beta_{4}\cdot {\mathrm{WeeklyArousal}}_{t}+\beta_{5}\cdot {\mathrm{WeeklyValence}}_{t}+\varepsilon \end{array}$$

The interaction models asked: Is the effect of breathing exercises on weekly stress moderated by how stressed the person felt the previous week and by their user experience?

(A4) $$\begin{array}{cc} {\mathrm{WeeklyStress}}_{t}= & \beta_{0}+\beta_{1}\cdot {\mathrm{WeeklyStress}}_{t-1}+\beta_{2}\cdot {\mathrm{WeeklyExercises}}_{t}+\beta_{3}\cdot \mathrm{UserExperience}\\ \; & +\beta_{4}\cdot {\mathrm{WeeklyArousal}}_{t}+\beta_{5}\cdot {\mathrm{WeeklyValence}}_{t}\\ \; & +\beta_{6}\cdot \left({\mathrm{WeeklyStress}}_{t-1}\times {\mathrm{WeeklyExercises}}_{t}\right)\\ \; & +\beta_{7}\cdot \left({\mathrm{WeeklyStress}}_{t-1}\times \mathrm{UserExperience}\right)\\ \; & +\beta_{8}\cdot \left({\mathrm{WeeklyExercises}}_{t}\times \mathrm{UserExperience}\right)\\ \; & +\beta_{9}\cdot \left({\mathrm{WeeklyStress}}_{t-1}\times {\mathrm{WeeklyExercises}}_{t}\times \mathrm{UserExperience}\right)+\varepsilon \end{array}$$

To examine whether the total amount of performed breathing exercises relates to examinational stress, we modeled examinational stress scores as a function of participants’ experience across the semester using cluster robust ordinary least squares (OLS) regression in Python 3.13 (statsmodels 0.15). For every participant, we first calculated the mean weekly stress (the average of their 30-item stress surveys over all the weeks they completed), ${\mathrm{TotalExercises}}_{semester}$ (the total number of days on which a breathing session was logged throughout the semester), the mean arousal, and the mean valence across the study. Because some participants filled in the exam stress survey more than once (i.e., they had more than one exam), we treated those rows as repeated measures, and we clustered all the standard errors on Participant; the point estimates are therefore identical to classical OLS, but *t*-tests and omnibus *F* take the within person correlation into account (Python 3.13—statsmodels 0.15, LBFGS optimizer).

The main effect models asked: Do people who perform more breathing exercises throughout the semester experience different examinational stress levels?

(A5) $$\begin{array}{c} \mathrm{ExamStress}=\beta_{0}+\beta_{1}\cdot \mathrm{MeanWeeklyStress}+\beta_{2}\cdot {\mathrm{TotalExercises}}_{semester}+\beta_{3}\cdot \mathrm{UserExperience}\\ +\beta_{4}\cdot \mathrm{MeanArousal}+\beta_{5}\cdot \mathrm{MeanValence}+\varepsilon \end{array}$$

The interaction models asked: Is the next day effect of breathing exercises on examinational stress moderated by how stressed the person felt throughout the semester and by their user experience?

(A6) $$\begin{array}{cc} \mathrm{ExamStress}= & \beta_{0}+\beta_{1}\cdot \mathrm{MeanWeeklyStress}+\beta_{2}\cdot {\mathrm{TotalExercises}}_{semester}+\beta_{3}\cdot \mathrm{UserExperience}\\ \; & \;+\beta_{4}\cdot \mathrm{MeanArousal}+\beta_{5}\cdot \mathrm{MeanValence}\\ \; & \;+\beta_{6}\cdot \left(\mathrm{MeanWeeklyStress}\times {\mathrm{TotalExercises}}_{semester}\right)\\ \; & \;+\beta_{7}\cdot \left(\mathrm{MeanWeeklyStress}\times \mathrm{UserExperience}\right)\\ \; & \;+\beta_{8}\cdot \left({\mathrm{TotalExercises}}_{semester}\times \mathrm{UserExperience}\right)\\ \; & \;+\beta_{9}\cdot \left(\mathrm{MeanWeeklyStress}\times {\mathrm{TotalExercises}}_{semester}\times \mathrm{UserExperience}\right)+\varepsilon \end{array}$$

All the models were estimated by maximum likelihood with an L-BFGS optimizer; fixed effect coefficients were evaluated via Wald *z*-tests. We report marginal and conditional $R^{2}$ and an omnibus Wald $\chi^{2}$ for the joint significance of all fixed effects.

## References

[B1-behavsci-16-00778] Balban M. Y., Neri E., Kogon M. M., Weed L., Nouriani B., Jo B., Snyder M. P., Spiegel D., Huberman A. D. (2023). Brief structured respiration practices enhance mood and reduce physiological arousal. Cell Reports Medicine.

[B2-behavsci-16-00778] Balters S., Mauriello M. L., Park S. Y., Landay J. A., Paredes P. E. (2020). Calm commute: Guided slow breathing for daily stress management in drivers. Proceedings of the ACM on Interactive, Mobile, Wearable and Ubiquitous Technologies.

[B3-behavsci-16-00778] Bentley T. G. K., D’Andrea-Penna G., Rakic M., Arce N., LaFaille M., Berman R., Kogon M., Sprimont P. (2023). Breathing practices for stress and anxiety reduction: Conceptual framework of implementation guidelines based on a systematic review of the published literature. Brain Sciences.

[B4-behavsci-16-00778] Bentley T. G. K., Seeber C., Hightower E., Mackenzie B., Wilson R., Velazquez A., Sprimont P., Lorenz K. A. (2022). Slow-breathing curriculum for stress reduction in high school students: Lessons learned from a feasibility pilot. Frontiers in Rehabilitation Sciences.

[B5-behavsci-16-00778] Bradley M. M., Lang P. J. (1994). Measuring emotion: The self-assessment manikin and the semantic differential. Journal of Behavior Therapy and Experimental Psychiatry.

[B6-behavsci-16-00778] Brinsley J., O’Connor E. J., Singh B., McKeon G., Curtis R., Ferguson T., Mellow M. L., Dumuid D., Szeto K., Simpson C. E., Maher C. (2025). Effectiveness of digital lifestyle interventions on depression, anxiety, stress, and well-being: Systematic review and meta-analysis. Journal of Medical Internet Research.

[B7-behavsci-16-00778] Buttazzoni A., Brar K., Minaker L. M. (2021). Smartphone-based interventions and internalizing disorders in youth: Systematic review and meta-analysis. Journal of Medical Internet Research.

[B8-behavsci-16-00778] Debowska A., Horeczy B., Boduszek D., Dolinski D., von Bastian C. C. (2022). Development and validation of a stress response measure: The Daily Stress Response Scale (DSRS). Health Psychology Report.

[B9-behavsci-16-00778] Farrall A., Taylor J., Ainsworth B., Alexander J. (2023). Manifesting breath: Empirical evidence for the integration of shape-changing biofeedback-based artefacts within digital mental health interventions *[Conference session]*. Proceedings of the 2023 CHI Conference on Human Factors in Computing Systems.

[B10-behavsci-16-00778] Fincham G. W., Strauss C., Montero-Marin J., Cavanagh K. (2023). Effect of breathwork on stress and mental health: A meta-analysis of randomised-controlled trials. Scientific Reports.

[B11-behavsci-16-00778] Finstad K. (2010). The usability metric for user experience. Interacting with Computers.

[B12-behavsci-16-00778] Gan D. Z., McGillivray L., Han J., Christensen H., Torok M. (2021). Effect of engagement with digital interventions on mental health outcomes: A systematic review and meta-analysis. Frontiers in Digital Health.

[B13-behavsci-16-00778] Garg P., Mendiratta A., Banga A., Bucharles A., Piccoli M. V. F., Kamaraj B., Sahu K. K., Kashyap R. (2024). Effect of breathing exercises on blood pressure and heart rate: A systematic review and meta-analysis. International Journal of Cardiology Cardiovascular Risk and Prevention.

[B14-behavsci-16-00778] Graves B. S., Hall M. E., Dias-Karch C., Haischer M. H., Apter C. (2021). Gender differences in perceived stress and coping among college students. PLoS ONE.

[B15-behavsci-16-00778] Hadi R., Valenzuela A. (2020). Good vibrations: Consumer responses to technology-mediated haptic feedback. Journal of Consumer Research.

[B16-behavsci-16-00778] Honinx E., Broes S., Roekaerts B., Huys I., Janssens R. (2023). Existing meditation and breathing devices for stress reduction and their incorporated stimuli: A systematic literature review and competition analysis. Mayo Clinic Proceedings: Digital Health.

[B17-behavsci-16-00778] Honinx E., Meys M., Broes S., Van Langenhoven L., Janssens R., Huys I., Gosseries O. (2025). The effectiveness and user preferences of two tactile breathing devices in reducing stress in stressed individuals: A mixed methods study. International Journal of Clinical and Health Psychology.

[B18-behavsci-16-00778] Hopper S. I., Murray S. L., Ferrara L. R., Singleton J. K. (2019). Effectiveness of diaphragmatic breathing for reducing physiological and psychological stress in adults: A quantitative systematic review. JBI Evidence Synthesis.

[B19-behavsci-16-00778] Laborde S., Allen M. S., Borges U., Iskra M., Zammit N., Dosseville F. (2022). Psychophysiological effects of slow-paced breathing at six cycles per minute with or without heart rate variability biofeedback. Psychophysiology.

[B20-behavsci-16-00778] Lange B., Flynn S., Chang C. Y., Rizzo A., Bolas M. (2011). Breathe: A game to motivate the adherence of breathing exercises. Journal of Physical Therapy Education.

[B21-behavsci-16-00778] Lemon C., Huckvale K., Carswell K., Torous J. (2020). A narrative review of methods for applying user experience in the design and assessment of mental health smartphone interventions. International Journal of Technology Assessment in Health Care.

[B22-behavsci-16-00778] Leyro T. M., Versella M. V., Yang M. J., Brinkman H. R., Hoyt D. L., Lehrer P. (2021). Respiratory therapy for the treatment of anxiety: Meta-analytic review and regression. Clinical Psychology Review.

[B23-behavsci-16-00778] Li T.-T., Wang H.-Y., Zhang H., Zhang P.-P., Zhang M.-C., Feng H.-Y., Lan Y., Sun Z.-G. (2023). Effect of breathing exercises on oxidative stress biomarkers in humans: A systematic review and meta-analysis. Frontiers in Medicine.

[B24-behavsci-16-00778] Linardon J., Cuijpers P., Carlbring P., Messer M., Fuller-Tyszkiewicz M. (2019). The efficacy of app-supported smartphone interventions for mental health problems: A meta-analysis of randomized controlled trials. World Psychiatry.

[B25-behavsci-16-00778] Linardon J., Firth J., Torous J., Messer M., Fuller-Tyszkiewicz M. (2024). Efficacy of mental health smartphone apps on stress levels: A meta-analysis of randomised controlled trials. Health Psychology Review.

[B26-behavsci-16-00778] Loo Gee B., Griffiths K. M., Gulliver A. (2016). Effectiveness of mobile technologies delivering ecological momentary interventions for stress and anxiety: A systematic review. Journal of the American Medical Informatics Association.

[B27-behavsci-16-00778] Mahalakshmi B., Ghemabhai K. M., Gottlieb A. S., Sivasubramanian N., Parthasarathy P. (2024). Deep breathing exercises in easing educational stress among Indian high school students. Bioinformation.

[B28-behavsci-16-00778] Matheus K., Vázquez M., Scassellati B. (2022). A social robot for anxiety reduction via deep breathing *[Conference session]*. 31st IEEE International Conference on Robot and Human Interactive Communication (RO-MAN).

[B29-behavsci-16-00778] Mestdagh M., Verdonck S., Piot M., Niemeijer K., Kilani G., Tuerlinckx F., Kuppens P., Dejonckheere E. (2023). m-Path: An easy-to-use and highly tailorable platform for ecological momentary assessment and intervention in behavioral research and clinical practice. Frontiers in Digital Health.

[B30-behavsci-16-00778] Morgan S. P., Lengacher C. A., Seo Y. (2025). A systematic review of breathing exercise interventions: An integrative complementary approach for anxiety and stress in adult populations. Journal of Holistic Nursing.

[B31-behavsci-16-00778] Mundo A. I., Tipton J. R., Muldoon T. J. (2022). Generalized additive models to analyze nonlinear trends in biomedical longitudinal data using R: Beyond repeated measures ANOVA and linear mixed models. Statistics in Medicine.

[B32-behavsci-16-00778] Opie J. E., Vuong A., Welsh E. T., Gray R., Pearce N., Marchionda S., Khalil H. (2024). Outcomes of best-practice guided digital mental health interventions for youth and young adults with emerging symptoms: Part I. A systematic review of socioemotional outcomes and recommendations. Clinical Child and Family Psychology Review.

[B33-behavsci-16-00778] Ortega-Donaire L., Álvarez-García C., López-Franco M., Sanz-Martos S. (2023). Effectiveness of guided breathing and social support for the reduction of pre-exam anxiety in university students: A factorial study. Healthcare.

[B34-behavsci-16-00778] Ovadia-Blechman Z., Tarrasch R., Velicki M., Chalutz Ben-Gal H. (2022). Reducing test anxiety by device-guided breathing: A pilot study. Frontiers in Psychology.

[B35-behavsci-16-00778] Perreau E., Belouahchi S., Castel D., Loup-Escande É. (2023). Impact of ecological momentary interventions on regulatory strategies of perceived stress at work: An exploratory study based on the application ’MON SHERPA’ used in an ecological context. Review of European Studies.

[B36-behavsci-16-00778] Rosenberg A., Hamiel D. (2021). Reducing test anxiety and related symptoms using a biofeedback respiratory practice device: A randomized control trial. Applied Psychophysiology and Biofeedback.

[B37-behavsci-16-00778] Rudnicki K., Schepers L., Rummens K., Joris G., Poels K. (2025). The effectiveness of brief meditation assisted with cardiac biofeedback on interoceptive accuracy, sensibility and awareness: A randomized controlled trial. Applied Psychophysiology and Biofeedback.

[B38-behavsci-16-00778] Seppälä E. M., Bradley C., Moeller J., Harouni L., Nandamudi D., Brackett M. A. (2020). Promoting mental health and psychological thriving in university students: A randomized controlled trial of three well-being interventions. Frontiers in Psychiatry.

[B39-behavsci-16-00778] Sîrbu V., David O. A. (2024). Efficacy of app-based mobile health interventions for stress management: A systematic review and meta-analysis of self-reported, physiological, and neuroendocrine stress-related outcomes. Clinical Psychology Review.

[B40-behavsci-16-00778] Smits M., Kim C.-M., van Goor H., Ludden G. D. S. (2022). From digital health to digital well-being: Systematic scoping review. Journal of Medical Internet Research.

[B41-behavsci-16-00778] Smyth A., Syrek C., Reins J. A., Domin M., Janneck M., Lehr D. (2018). User experience predicts the effectiveness of a gamified recovery app: Investigation of Holidaily—An app promoting recovery behavior after vacation and during daily working life. Prävention und Gesundheitsförderung.

[B42-behavsci-16-00778] Strehli I., Burns R. D., Bai Y., Ziegenfuss D. H., Block M. E., Brusseau T. A. (2021). Mind–body physical activity interventions and stress-related physiological markers in educational settings: A systematic review and meta-analysis. International Journal of Environmental Research and Public Health.

[B43-behavsci-16-00778] Sung Y. T., Chao T. Y. (2015). Construction of the examination stress scale for adolescent students. Measurement and Evaluation in Counseling and Development.

[B44-behavsci-16-00778] Thayer J. F., Åhs F., Fredrikson M., Sollers J. J., Wager T. D. (2012). A meta-analysis of heart rate variability and neuroimaging studies: Implications for heart rate variability as a marker of stress and health. Neuroscience & Biobehavioral Reviews.

[B45-behavsci-16-00778] Toussaint L., Nguyen Q. A., Roettger C., Dixon K., Offenbächer M., Kohls N., Hirsch J., Sirois F. (2021). Effectiveness of progressive muscle relaxation, deep breathing, and guided imagery in promoting psychological and physiological states of relaxation. Evidence-Based Complementary and Alternative Medicine.

[B46-behavsci-16-00778] Waters L., Reeves M., Fjeldsoe B., Eakin E. (2012). Control group improvements in physical activity intervention trials and possible explanatory factors: A systematic review. Journal of Physical Activity and Health.

[B47-behavsci-16-00778] Wu A., Scult M. A., Barnes E. D., Betancourt J. A., Falk A., Gunning F. M. (2021). Smartphone apps for depression and anxiety: A systematic review and meta-analysis of techniques to increase engagement. NPJ Digital Medicine.

[B48-behavsci-16-00778] You M., Laborde S., Zammit N., Iskra M., Borges U., Dosseville F. (2021). Single slow-paced breathing session at six cycles per minute: Investigation of dose-response relationship on cardiac vagal activity. International Journal of Environmental Research and Public Health.

[B49-behavsci-16-00778] Zaccaro A., Piarulli A., Laurino M., Garbella E., Menicucci D., Neri B., Gemignani A. (2018). How breath-control can change your life: A systematic review on psycho-physiological correlates of slow breathing. Frontiers in Human Neuroscience.

